# Tailoring Crystallization Kinetics in Thin Sucrose Films during Convective Drying: Impact of Temperature and Humidity on Onset, Growth, and Nucleation Rate

**DOI:** 10.3390/pharmaceutics16101260

**Published:** 2024-09-27

**Authors:** Martin Schugmann, Petra Foerst

**Affiliations:** Food Process Engineering, School of Life Sciences, Technical University of Munich, Weihenstephaner Berg 1, 85354 Freising, Germany; petra.foerst@tum.de

**Keywords:** thin film drying, crystallization, nucleation, growth, humidity, sucrose

## Abstract

Drying experiments with varying air temperature and humidity were conducted to investigate the influence of the drying process on the crystallization of thin sucrose films. For the first time, the effects of the nucleation onset, nucleation rate, and growth rate were investigated in situ and their differentiated influence on product crystallinity could be assessed. The growth rate was not influenced by air humidity but showed a strong dependence on temperature. It increased with drying temperature; however, at high temperatures, growth was inhibited when the water content falls below a critical level. Noticeable differences in nucleation behavior could be observed with regard to air humidity. Dry air led to crystallization onsets at lower levels of supersaturation, while moderately humid air retarded it. At higher temperatures, nucleation onset commenced at lower water contents but at a constant supersaturation level. The nucleation rate doubled in experiments with moderately humid air (15% RH), while an elevated drying temperature showed generally lower nucleation rates. The observed differences in the nucleation onset and rate could be explained by the film-internal concentration profile, which is strongly influenced by drying parameters. The insights therefore provide a differentiated understanding of the formation of the physical state and how it can be influenced during convective drying.

## 1. Introduction

Convective drying of sugar-rich substances is widely employed in the pharmaceutical and food industries for the transformation of thermally sensitive feed solutions into powders. Industrially, often carried out by spray drying, it is utilized to enhance shelf life, encapsulate compounds such as active pharmaceutical ingredients, and improve the solubility of the final products [1,2,3]. The physical state of these products—crystalline or amorphous (or a mixed form with a specific degree of crystallinity)—is determined during or after drying. It is economically highly relevant due to the different, associated product properties. While minor components such as microorganisms, active pharmaceutical ingredients, or vitamins are well stabilized in an amorphous structure, thus ensuring high bioavailability and biochemical stability, their hygroscopicity makes them prone to caking, sticking, or post-crystallization under unfavorable storage or handling conditions [3,4]. Crystalline particles, on the other hand, offer better physicochemical stability during storage at elevated moisture or temperature as these particles do not undergo phase transition. Moreover, the crystallinity and crystallization behavior has a significant impact on morphology and the flowability of formed particles [5].

In order to avoid caking of the product to a large extent, sugar-rich powders can either be provided with caking-reducing additives [6,7,8] or be subjected to controlled post-crystallization in a fluidized bed [9,10,11]. However, the usage of additives is often avoided and post-crystallization in a fluidized bed is energy-intense as well as technically expensive. Great importance is therefore attached to the targeted control of the physical state (and thus the product properties) in situ during convective drying. Nevertheless, the influence of drying conditions on crystallization during the drying process is not yet fully understood and is the object of various research efforts, mainly focusing on the parameters air temperature and humidity [12,13,14,15,16].

The work of Hartel and his group has significantly advanced the understanding of crystal growth mechanisms and the influence of processing parameters on crystal formation and led to strategies in controlling crystal size and morphology. The focus of their work was mainly on the investigation of crystallization at constant initial concentrations [17] or on the heterogeneous nucleation by seeding during drying [18]. Thus, they did not investigate homogeneous nucleation during drying nor the crystallization onset or the nucleation rate.

In several publications, Woo and his group demonstrated the significant influence of humidity and temperature control on in situ crystallization in spray drying experiments. The importance of drying history on the crystallinity of the product was emphasized and the findings provide evidence about the existence of a critical period for crystallization. They found that in this intermediate stage of the drying process, in which the particle formation takes place, an extension of the critical period by the use of humid air results in a significant increase in the degree of crystallinity of the product [14,16]. However, the results were limited to post-drying DSC and XRD investigations of the spray-dried bulk and the exact progress and distinction between nucleation and crystal growth were not yet observed in situ [19].

With regard to controlling the degree of crystallinity via temperature, different approaches have been attempted to increase the crystallization rate during spray drying with elevated drying air temperatures by Islam and Langrish. Their main conclusion was that an elevated drying air temperature results in a large T − T_g_ (glass transition temperature) value, representing a substantial driving force for the solid-state crystallization [20,21]. Similar results were obtained by the use of high drying air temperature by Fu et al. for single droplet drying of lactose solutions at 70 and 110 °C [22]. The results indicate the existence of a critical drying period where rapid crystallization occurs and substantiate the above-mentioned intermediate stage transition crystallization theory. Fu et al. therefore postulate higher drying temperature and longer exposure time to the critical crystallization stage as ways leading to a high crystallinity. However, the concept of T − T_g_ as the driving force of crystallization does not distinguish between the different aspects of the formation and growth of crystals in the different phases of drying.

So far, little attention has been paid to the development of crystal growth and especially the nucleation behavior during the different stages of drying as well as in dependence on the drying conditions such as temperature and relative humidity. On the one hand, this is due to the generally difficult observability of phenomena on the size scale of spray dryer droplets and, on the other hand, due to complications in the observation of growing crystals in an amorphous matrix per se.

In the present study, the crystallization behavior of a sucrose solution was therefore examined in detail with the aid of a thin-film dryer. With this device, drying is possible under highly defined conditions regarding air temperature, humidity, and air velocity with the particular possibility to independently control these parameters and also with accurate inline water content determination [23]. The comparatively long time scale in which crystallization may occur during thin-film drying also allows a study of the crystallization in detail due to the slower drying kinetics. Furthermore, the setup allows for a contactless analysis of nucleation and growth phenomena in situ without biasing the results by a time shift or changes in temperature or humidity, inherent in offline analyses. This allows for improved interpretability of the crystallization behavior.

This work is intended to contribute to a better understanding of the crystallization of amorphous sucrose during convective drying and focuses on the experimental in situ investigation of the nucleation onset, nucleation rate, and crystal growth rate by independently varying the main drying parameters air temperature and drying air relative humidity.

## 2. Materials, Equipment, and Methods

### 2.1. Preparation of Solution

For all experiments, a solution of ultrapure water (MilliQ Direct; Merck Darmstadt, Germany) and sucrose (purity ≥ 99.7%; Carl Roth GmbH, Karlsruhe, Germany) with a water content on a dry basis of 9 g*g^−1^ (*w*/*w*) was prepared in a 100 mL volumetric flask with a ground glass stopper, mixed thoroughly several times and left to stand at 5 °C for 24 h. To avoid contamination, the solutions were not used for longer than one day. The volumetric flask was warmed to room temperature before the experiment was carried out. All further indications of the water content refer to the water content on a dry basis.

### 2.2. Thin-Film Drying

Drying was carried out in a humidity-, temperature-, and airflow-controlled thin-film dryer, the design, operation, and validation of accuracy of which are described in detail in [23]. The drying air used was cleaned of particles by using a fine filter and activated carbon filter (Drypoint; Beko Technologies GmbH, Neuss, Germany) to avoid heterogeneous nucleation on the film surface to the greatest possible extent. Preliminary experiments validated that the filters effectively inhibit heterogeneous nucleation to an extent where it is negligible. In total, 1.7 g of the sucrose solution was pipetted into a 0.07 mm deep square cavity of 25 cm^2^ on an aluminum sample platelet (52 mm × 52 mm × 1 mm) and inserted into the drying channel. The drying kinetics was recorded using a precision balance (Pioneer PX225e; Ohaus Europe GmbH, Nänikon, Switzerland, reproducibility: 0.1 mg). Details of the method for increased gravimetric accuracy and the prevention of condensation phenomena are described in [23]. After drying, the platelet was taken out and reweighted to verify the drying kinetics’ accuracy. To guarantee a uniform thickness of the film, high care was taken for leveling the sample and the evenness was verified after each experiment with the aid of a micrometer screw. All drying experiments were conducted in triplicate and at a drying air velocity of 0.3 m*s^−1^. The drying air temperatures and humidities (relative humidities and corresponding humidity ratios) used are listed in Table A1 in Appendix A.

Besides the experiments at constant drying air parameters, further exemplary experiments were conducted at 80 and 100 °C with constant temperature but in situ decreasing drying air humidity. For this purpose, the solution was first dried to the equilibrium water content with a drying air humidity of 40% RH. Then, the humidity of the drying air was slowly reduced in 2% steps during the experiments. The equilibrium water content at each humidity level was held for at least 30 min to guarantee sufficient concentration equilibration inside the film.

The supersaturation S mentioned in the results is defined as the quotient of the saturation water content and the experimentally measured water content. The water content of saturation refers to the polynomial models for sucrose saturation by Charles and Vavrinecz in their corresponding areas of application [24,25].

### 2.3. Analyzation of Nucleation and Growth

**Figure 1:**
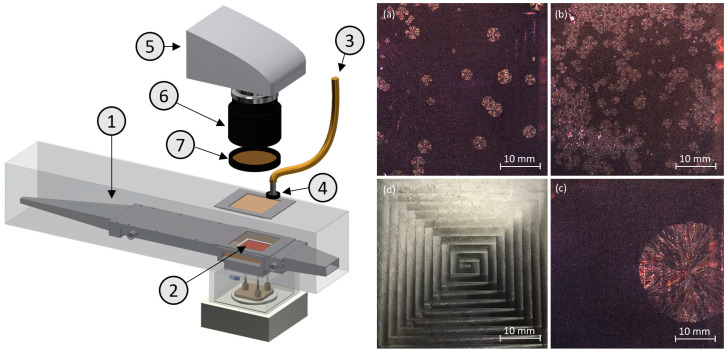
(**Left**): Schematic measurement setup for determination of nucleation onset, nucleation rate, and growth rate: (1) drying channel with windows, (2) thin film on platelet, (3) flexible light guide of halogen light source, (4) light polarizing filter, (5) camera, (6) zoom lens, and (7) camera polarizing filter. (**Right**): Exemplary partially crystallized thin films at drying air temperature of 80 °C and different relative humidities of drying air of (**a**) 0%, (**b**) 15%, and (**c**) 30% RH under polarized light and (**d**) under unpolarized light and with untreated platelet surface.

A schematic overview of the experimental setup with the drying channel and the equipment for the observation of nucleation and growth is drafted on the left-hand side of Figure 1. To make nucleation and growth visible, a halogen light source with a flexible light guide (KL 1500 LCD; Schott Glas Fiber Optics Division, Mainz, Germany) was placed above the drying channel and the light was polarized using an optical filter (XP40HT-40; Edmund Optics, Mainz, Germany). The thin film was observed through glass planes in the drying channel using a camera (Leica EC4; Leica Microsystems GmbH, Wetzlar, Germany, resolution: 13 µm), a zoom lens (Pentax Cosmicar TV 12.5–75 mm; Pentax Ricoh Imaging GmbH, Hannover, Germany), and a rotatable polarizing filter (Olympus U-POT Polarizer; Evident Europe GmbH, Hamburg, Germany), which was placed in front of the lens. The polarizing filter was rotated for the observation in such a way that the transmission in the amorphous area of the thin film was minimized and the crystals formed were visible in good contrast by their optical activity. To reduce reflections, the surface of the milled cavities on the sample holding platelet was treated with 4 mL of 10% HCl, for 20 min. Images (a) to (c) on the right-hand side of Figure 1 show partially crystallized thin films for different drying conditions under the polarized light and treated surface whereas image (d) exemplarily shows an unetched, reflecting platelet (not used) under unpolarized light. Preliminary, comparative experiments with unetched and etched platelets showed that the surface treatment had no effect on crystallization behavior.

The exact moment of nucleus formation cannot be directly observed with a camera due to limited resolution and the nucleation’s nanoscale nature. Assuming constant crystal growth thereafter, the exact point of formation was back-calculated with the determination of the observable crystal size as similarly performed for oversaturated organic solutions by Jiang et al. [26] or for sugar glasses by Levenson et al. [17]. However, taking the observed growth rates into account (see Section 3.3), the time of the nuclei to grow into a well-observable size (50 µm) was considered negligibly short compared to the general drying time.

Once nucleation started, the number of crystals was counted to calculate the nucleation rate. For this purpose, an image of the drying film was taken every 60 s with the aid of an Arduino Micro (Arduino S.r.l., Scarmagno, Italy). The number of newly formed crystals was evaluated using the counting function of the image processing software ImageJ 1.53. The nucleation rate was defined as the number of nucleation events in the observed thin-film volume per time [s^−1^*m^−3^]. The nucleation was related to the remaining amorphous film volume, which decreases during progressive drying. The volume of the drying film was determined on the basis of the post-drying determination of the film thickness (which was about 50 µm) via microscopy (VHX-7000; Keyence Deutschland GmbH, Neu-Isenburg, Germany) and a micrometer screw and the assumption of ideal shrinkage, which applies in good approximation to concentrated sugar thin films [27]. However, the volume change turned out to be negligibly small between the nucleation onset and the end of the experiment due to low evaporation rates in this period. Furthermore, the growth rate is defined as the change in crystal radius per time [µm*min^−1^]. The radius was measured in four directions per crystal using ImageJ (length measurement function) and the mean value was recorded. To determine the growth rate, the change in size was recorded for two subsequent images (60 s), for low growth rates below 50 µm*s^−1^ over a period of 120 s. The standard time interval of 60 s proved to be a good compromise between resolution and the change in the growth rate due to progressive drying during this period. The growth rate was measured on crystals forming spontaneously during drying.

### 2.4. Differential Scanning Calorimetry

To verify the physical state of the crystalline and amorphous regions, samples of these areas were examined using dynamic and isothermal differential calorimetry (DSC 214 Polyma; NETZSCH-Gerätebau GmbH, Selb, Germany). For this purpose, 5–15 mg of the thin-film material was taken at the corresponding point, directly transferred into 60 µL aluminum crucibles (Concavus pan; NETZSCH-Gerätebau GmbH, Selb, Germany), which were subsequently cold-welded and analyzed. The samples then were heated to 120 °C at a heating rate of 10 °C*min^−1^ and held for 60 min.

## 3. Results and Discussion

### 3.1. Validation of Crystalline and Amorphous State by Differential Scanning Calorimetry

**Figure 2:**
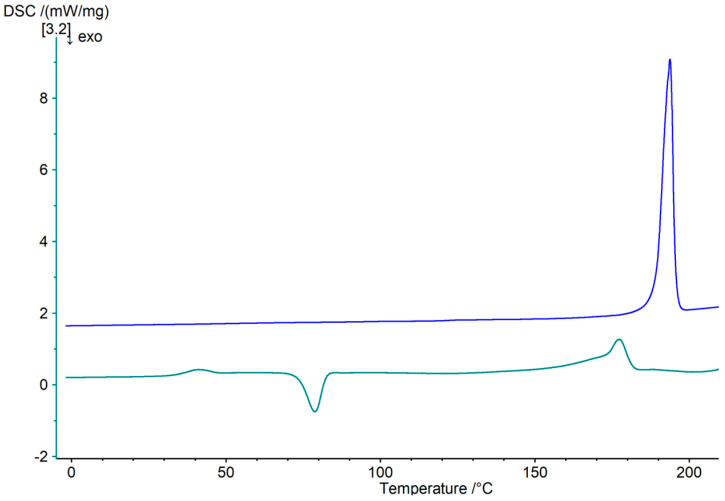
DSC thermogram of crystalline (blue) and amorphous (cyan) part of dried sucrose film. Sample mass was 5–15 mg and heating rate was 10 °C*min^−1^.

In the experiments, the samples were analyzed visually with the aid of the specific interaction of crystalline areas with polarized light as described in the previous section. Crystalline sucrose shows optical activity and rotates the plane of polarization; hence, crystalline areas become visible through a polarizing filter, while the reflected light of amorphous structures is blocked by the filter. On this basis, crystal regions were identified. To validate this analyzation method, each sample of an apparently amorphous and a crystalline area were measured in the DSC. For all examined crystalline areas, the DSC analysis showed an endothermal melting peak without prior endothermal glass transition and without subsequent recrystallization phenomena in the isothermal phase (see Figure 2). This confirmed the complete crystallization of the according thin-film area, whereas amorphous samples showed a typical endothermal step, denoting glass transition and/or exothermal recrystallization peaks afterwards. From the clear glass transition and the melting peak in the same dimension as the previous recrystallization peak, it can be concluded that the sample consisted of amorphous material.

### 3.2. Drying Kinetics

**Figure 3:**
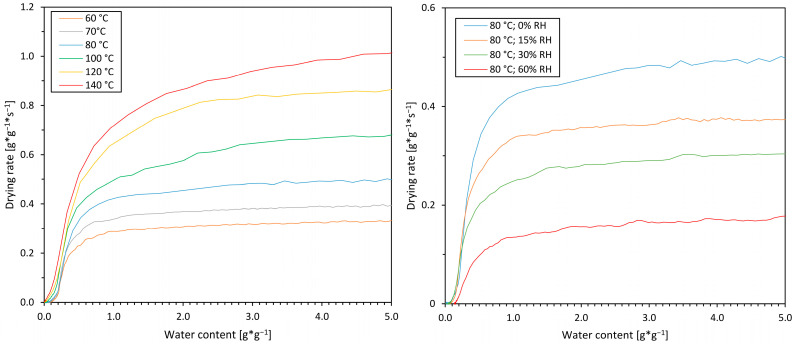
(**Left**): Thin-film drying kinetics of the experiments for sucrose drying at drying air temperatures of 60, 70, 80, 100, 120, and 140 °C. Drying was conducted with anhydrous air and an air velocity of 0.3 m*s^−1^. (**Right**): Thin-film drying kinetics of the experiments for sucrose drying at a drying air temperature of 80 °C and relative humidities of 0, 15, 30, and 60% RH and a drying air velocity of 0.3 m*s^−1^. Initial water content was 9 g*g^−1^.

The drying kinetics for experiments with varying drying air temperatures and dry air is shown on the left-hand side of Figure 3, while the drying kinetics for different air humidities at a constant temperature of 80 °C is displayed on the right-hand side.

All experiments exhibited a typical constant rate period after the start of drying at a water content of 9 g*g^−1^, which continued up to a water content of about 1 g*g^−1^ before switching to the falling rate period. For higher temperatures, the transition between the constant and falling rate was less distinctive and began at a slightly higher water content. An increased drying air temperature had a positive effect on the drying rate, while an increasing humidity level reduced the drying rate.

### 3.3. Crystal Growth at Different Air Temperatures and Humidity

#### 3.3.1. Drying Air Temperature

**Figure 4:**
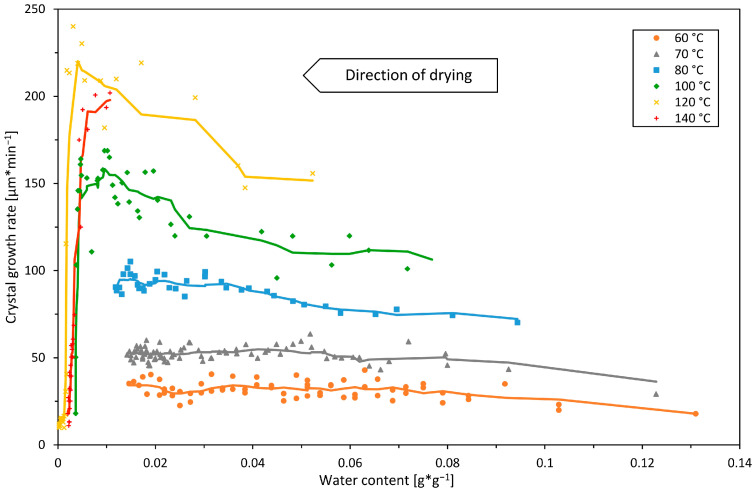
The crystal growth rate during drying of sucrose at temperatures between 60 and 140 °C. The individual points refer to the evaluation of a single crystal front in the observed period of 60 s. The lines refer to the moving average and serve to guide the eye. Initial water content was 9 g*g^−1^ and the sample mass was 1.7 g. The humidity of the air was 0% RH at a velocity of 0.3 m*s^−1^.

The growth rate was investigated at constant temperatures between 60 °C and 140 °C. Figure 4 shows the temperature dependency of the growth rate during drying as a function of the water content for dry air and an air velocity of 0.3 m*s^−1^.

The growth rate generally increases strongly with the drying temperature. It is in the range of 20 to 40 µm*min^−1^ for the drying experiments at 60 °C and reaches maximum values of almost 250 µm*min^−1^ for the experiments at 120 °C. The growth rate also rises with the ongoing drying: First, an increase in the growth rate can be observed at all drying temperatures with the ongoing decrease in water content. This dependence of the water content on the growth rate is generally more pronounced at higher temperatures, recognizable in the initially steeper course after the crystallization onset. As drying progresses, the concentration, and thus the supersaturation and hence the driving force for the crystallization of the sugar film, increases. In this regard, Mathlouthi and Genotelle state that sucrose crystal growth involves at least two key stages at the molecular level: first, the diffusion of sucrose from the bulk solution to the boundary layer at the crystal–solution interface, and second, the integration of sucrose molecules into the crystal lattice, which occurs after the molecules shed their hydration water [28]. Our findings suggest that viscosity in this area of water content is a relatively minor obstacle, whereas the detachment of hydration water from the sucrose molecules constitutes a major barrier. The second is favored by the removal of water during drying.

However, high air temperatures above 100 °C led to a rapid inhibition of the growth at water contents of around 0.01 g*g^−1^. This reduction in the growth rate stems from the exceedingly high viscosity and limited molecular movement inherent in highly concentrated solutions and is in line with the findings of Hartel [13] and Chen et al. [29], who observed a similar reduction in growth rates in sucrose–corn syrup mixtures above concentrations of 95%. At lower temperatures, however, respectively, at 60, 70, and 80 °C, no such growth rate inhibition could be observed. Therefore, our experimental data suggest that the water content during drying is always in a range at which the sugar concentration is not yet so high that growth is distinctively restricted by viscosity.

#### 3.3.2. Drying Air Humidity

**Figure 5:**
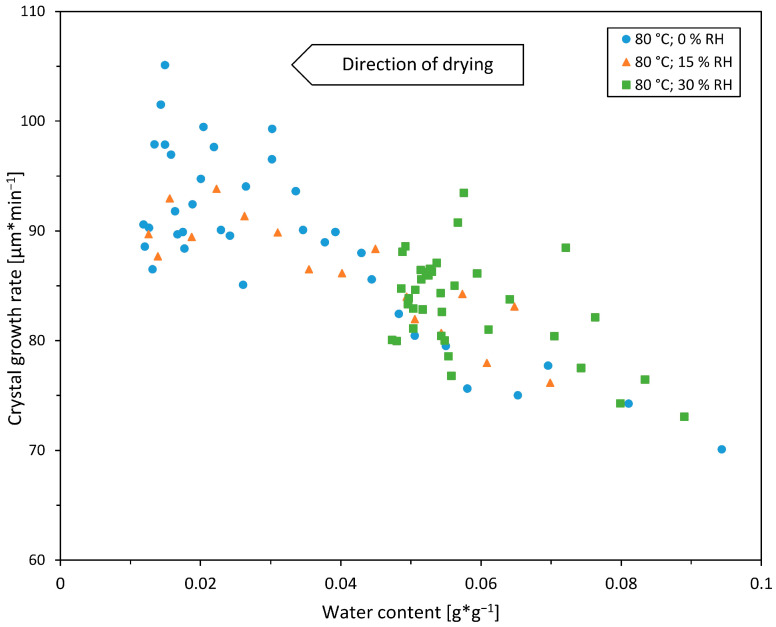
Crystal growth rates for a drying temperature of 80 °C and at three different air relative humidities of 0%, 15%, and 30% RH. Drying with a relative humidity of 60% did not show crystallization. The velocity of drying air was 0.3 m*s^−1^.

Furthermore, crystal growth rates were examined at 80 °C for different air humidities at levels of 15%, 30%, and 60% RH. The results are shown in Figure 5 and showed that the humidity did not affect the crystal growth rate at this temperature. For the same experiment at 60% RH, no nucleation occurred and the growth rate was therefore not measured. Consequently, it can be concluded that air temperature is the main influencing factor on the growth rate as sucrose crystallization exclusively commenced in the second drying period (compare Figure 3). Here, the product temperature matches the temperature of the drying air [30]. Temperature as the key parameter for growth can be explained by its effect on the diffusion via the diffusion coefficient. Other than in highly diluted systems, the diffusion coefficient in viscous systems is generally described as an exponential, Arrhenius-like function of the temperature, explaining its great dependency on growth [30,31]. In contrast to drying air temperature, humidity has no influence on the product temperature and thus the diffusivity in the carbohydrate matrix, as effects due to possible differences of the wet-bulb temperature can mainly be excluded in the period of crystallization.

However, the humid drying air clearly affects the drying rate (see right-hand side of Figure 3). The deceleration of drying hence generally extends the period of time in which growth is possible and thus affects total crystallinity after drying. With regard to the post-drying degree of crystallinity of spray-dried lactose, Islam et al. investigated the influence of elevated humidity by the recirculation of drying air with the effect of a higher post-drying degree of crystallinity for the particles compared with the product of a conventional spray drying setup [32]. They concluded that high humidity on the one hand increased the degree of crystallinity by keeping the particles’ glass transition low, thus enhancing molecular mobility and crystallization. Also, they came to the conclusion that increased humidity leads to higher wet-bulb temperatures and consequently higher product temperatures, which they assumed promoted crystallization. The fact that our experimental growth rates are comparable at different humidity levels (but at the same temperature) narrows down the probable causes of the altered degree of crystallinity reported by Islam et al.: it leads to the conclusion that the higher degree of crystallinity was probably less due to the lowering of the glass transition temperature by means of crystal growth as a lower glass transition, and thus a higher water content results in slower growth rates. It likely originated from the positive effect on the growth rate of a higher product (resp. wet bulb) temperature in the first drying period or a premature crystallization onset and hence the extension of the time phase during drying in which growth is possible. With regard to the extension of the period of crystal growth, experimental data on the nucleation onset and associated discussion can be found in the subsequent section. In conclusion and from the point of view of process control, our experimental results suggest that the product temperature is the main command variable for influencing growth during sucrose drying, and presumably generally for products, which show crystallization in the second drying stage. As the air humidity affects the wet-bulb and therefore the products’ temperature in the first drying stage, growth may be controlled here by drying air humidity, whereas during the second drying stage, as product temperature approximates air temperature, growth is solely controllable by air temperature.

### 3.4. Nucleation at Different Air Temperatures and Humidity

#### 3.4.1. Drying Air Humidity

Experiments were carried out at four different humidity levels to investigate the influence of drying air humidity on the nucleation behavior.

**Figure 6:**
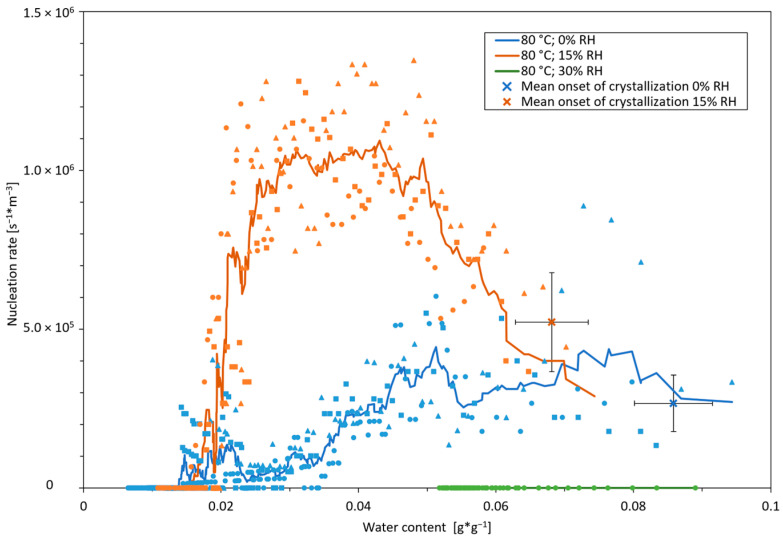
Nucleation rates and onsets for thin-film drying of sucrose solution at 80 °C at three different air relative humidities of 0%, 15%, and 30% for 80 °C. Initial water content was 9 g*g^−1^ and sample mass was 1.7 g. Drying air velocity was 0.3 m*s^−1^. Different symbols denote different experiments; error bars denote mean absolute errors.

Figure 6 shows the measured nucleation rates and nucleation onset at a relative humidity of 0%, 15%, and 30% RH during drying at 80 °C. The experiments at 60% RH showed no nucleation and are therefore not displayed.

During drying with anhydrous air, the nucleation onset commenced at a water content of X = 0.086 ± 0.006 g*g^−1^. While drying with moderately humid air of 15% RH, nucleation started at a considerably lower water content of X = 0.068 ± 0.005 g*g^−1^. Drying at 30% RH, only one of the three experiments showed crystallization with an onset at X = 0.089 g*g^−1^, and no crystallization was observed in the experiments at 60% RH, resulting in a nucleation rate of 0 in both cases. In view of these values for the crystallization onset and taking into account the drying kinetics (Figure 3), it is clear that crystallization consistently occurred at water contents well after the constant rate period (ending at about 1 g*g^−1^) in all experiments. Here, evaporation rates are low, which allows for an approximation of the surface temperature to the drying air temperature [30]. For thin-film (and spray) drying processes, it is furthermore typically assumed that there are small Biot numbers and, consequently, uniform temperatures prevail throughout the entire material [33]. Therefore, during the period of crystallization, it can be assumed that the product temperature corresponds to the drying air temperature for all shown results.

During drying with anhydrous air, the nucleation started at rates of about 2.7 × 10^5^ ± 0.9 × 10^5^ s^−1^m^−3^, remains at this level up to a water content of about 0.045 g*g^−1^, and decreases more or less steadily in the further course to come to a halt at a water content of about 0.015 g*g^−1^. When drying with moderately humid air of 15% RH, nucleation starts at a considerably higher nucleation rate of 5.2 × 10^5^ ± 1.6 × 10^5^ s^−1^m^−3^ and then rapidly increases to about twice this level. This rate is maintained over a wide water content range and is more than twice as high as the maximum values during drying with anhydrous air. Below 0.03 g*g^−1^, the nucleation rate drops abruptly and comes to a halt in the range of 0.015 g*g^−1^ just as in the case of the experiments with anhydrous air. When drying at 30% RH, there were only three nucleation events (in total) detected in one of the three experiments, and no crystal formation was observed in the experiments at 60% RH, resulting in a nucleation rate of 0 in both cases. Nevertheless, these events are also shown in Figure 6 to obtain an impression about the corresponding range of water content. The lack of considerable nucleation is most likely due to equilibrium water contents that are in a zone of supersaturation, but too weakly supersaturated to achieve relevant nucleation in the time scale of thin-film drying, further mentioned and discussed as the metastable zone. It can generally be noted that in the area of progressed drying below 0.015 g*g^−1^, the formation of new nuclei—at least via homogeneous nucleation—is already inhibited, but crystal growth is not yet inhibited (see Figure 4). The extent of growth and nucleation results from the interplay of the (physico-)chemical potential of supersaturation (in relation to the thermodynamic equilibrium resp. state of saturation) as driving force, which increases with the concentration, and the equally increasing viscosity as a measure of the mobility of the molecules as the inhibiting force. The fact that nucleation comes to a standstill in the same range of water content at different drying air humidities may, just as at the end of growth too, be due to the high viscosity in the thin film. The diffusion-limiting effect thus increasingly inhibits nucleation despite the high physicochemical potential. In summary, while the growth rate during drying is mainly determined by the drying temperature, and air humidity had no impact, significant differences in nucleation behavior were found for drying at different air humidities. With regard to the water content, the differences concern both the concentration at the onset of crystallization as well as the extent, i.e., the nucleation rate.

The influence of air humidity has been studied in relatively little detail to date, although it seems to have a major influence. Besides the already mentioned positive impact of humidity on the degree of crystallinity by Islam et al. [32], Shakiba et al. observed the same effect by the manipulation of the humidity milieu in a spray column via the counter-current mode of operation [34]. In continuing experiments, they artificially extended the mean time of particles in a concentrated, yet still wet, state (“intermediate drying stage”) by the injection of humidity at certain heights, which had a large effect on crystallinity of the dried product [14]. Here, also, solely the crystallinity of the bulk was examined after drying, making it impossible to precisely determine the mechanistic impact of growth and nucleation on the in situ crystallization process. Regarding the crystallization-favorable effect of humidity during drying, Shakiba’s results align with our observations of the nucleation behavior in this study and the results can be extended with regard to the origin of the increase in crystallinity: drying with slightly humid air (15% RH) allows high supersaturation values but still sufficient molecular mobility for the onset of nucleation as a basis for crystallization. The considerably higher nucleation rates over a wide range of water content have a positive influence on crystallization. Additionally, the relevant water content range of high nucleation occurrence can be attributed to the above-mentioned intermediate drying stage proposed by Shakiba. Apart from the prolonged crystallization time due to slower drying, our results target this finding to a substantial increase in the nucleation rate as a probable cause, on the one hand, and narrow down the existence of relevant stages for inline crystallization, which can be influenced by the drying kinetics, on the other hand.

#### 3.4.2. Drying Air Temperature

**Figure 7:**
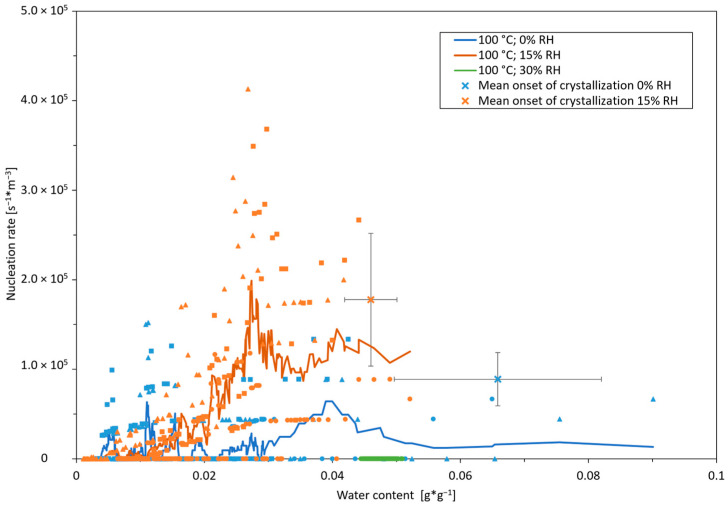
Nucleation rates and onsets for thin-film drying of sucrose solution at 100 °C at three different air humidity ratios, x_air_ of 0, 0.111, and 0.270 g*g^−1^. Initial water content was 9 g*g^−1^ and sample mass was 1.7 g. Drying air velocity was 0.3 m*s^−1^. Different symbols denote different experiments; error bars denote mean absolute errors.

To investigate the nucleation behavior at a different temperature level, it was further investigated by means of experiments at 100 °C at three different air humidity ratios, x_air_ of 0, 0.111, and 0.270 g*g^−1^. The onset of nucleation and nucleation rate are depicted in Figure 7.

The drying experiments for different air humidities at 100 °C show a qualitatively similar picture at 80 °C, but with generally lower nucleation rates and higher relative deviation. Just as in the experiments at 80 °C, nucleation in moderately humid air (air humidity ratio x_air_ of 0.11 g*g^−1^) starts only at a lower water content (higher supersaturation), but then at higher rates than in dry air. However, the deviation of the nucleation onset, depicted by the mean absolute errors, is also larger for the drying at 100 °C, leading to a less clear, but still noticeable, difference between the different humidities. It can also be noted that for the drying temperature of 100 °C, the formation of new nuclei ends just below 0.01 g*g^−1^ less distinctively and at a lower water content than for 80 °C. In this area of very high supersaturation (compare Figure 7), molecular mobility appears to be facilitated by the higher temperature to such an extent that nucleation still occasionally occurs. The nucleation rate for 100 °C is considerably lower than for 80 °C. This may be explained on a theoretical, molecular basis by the temperature dependence of the critical nucleus size as reflected, for instance, in the classical nucleation theory. With higher temperatures, larger molecule clusters are required for nucleus stability [35]. As this is less likely to occur, it leads to a general lower nucleation rate. The effect of the drying temperature on the crystallization of spray-dried goods has already been investigated for selected sugar-rich systems. Chiou et al. found that the higher the drying temperature, the higher the degree of crystallization [20], however, without distinction between the different aspects of crystallization, namely the growth, nucleation onset, and nucleation rate. This result was subsequently confirmed by further experiments on high-temperature spray drying by Islam and Langrish [21]. They conclude that a high drying air temperature leads to a large difference between the glass transition temperature and the particles’ temperature, which in turn is a major driving force for solid-state crystallization. Our experiments clearly show that the influence of nucleation on the crystallization decreases as the drying temperature increases due to substantially lower nucleation rates. The higher crystallinity by an increase in drying temperature reported by Chiou et al. is thus possibly achieved through much higher growth rates, which compensate for the lower total nucleation count and the later crystallization onset.

#### 3.4.3. Spatial Explanation Approach for the Nucleation Behavior

**Figure 8:**
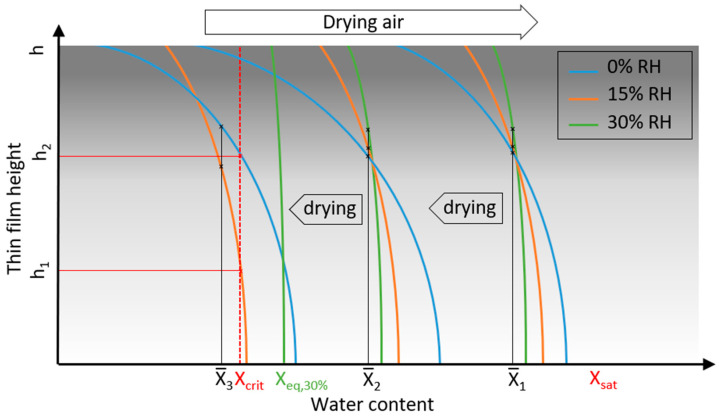
The film-internal, vertical water content profile as an explanation approach to the nucleation onset and progress of the nucleation rate during convective drying: fast drying with anhydrous air generates higher concentration differences between the surface and deeper layers, whereas slower drying allows for more pronounced diffusive equalization (blue line). In an early state of drying (average water content ${\bar{X}}_{1}$), the saturation water content (X_sat_) is exceeded but is too low to allow crystallization for all drying air humidities. During further drying (to a mean water content, ${\bar{X}}_{2}$), nucleation commences for the film dried with anhydrous air, as it exceeds critical water contents (X_crit_) in the top layers. Nucleation for the film dried with slightly humid air (orange line) commences, delayed, at an average water content above ${\bar{X}}_{2}$. With further drying, compared at the same average water content ${\bar{X}}_{3}$, nucleation proceeds at comparatively high rates, as X_crit_ is exceeded with a higher thin-film share (h − h_1_) than drying with anhydrous air (h − h_2_). Drying at high humidities (green line) leads to an equilibrium water content (X_eq,30%_) higher X_crit_. The same concept explains the observed abrupt inhibition of nucleation at very low water contents for experiments with slightly humid air.

Yet, of particular interest is the explanation of the cause of the strong observed influence of drying conditions on the nucleation rate and onset. At concentrations above the metastable zone, homogenous nucleation can occur in principle and, as observed, the extent of nucleation (and growth) depends on the prevailing supersaturation. However, concentration is not uniformly distributed inside the drying film due to the rapid drying process but there are differences between the film surface, which is subject to moisture transfer to the air, and the deeper layers with higher water contents. During progressed drying, this results in a more or less pronounced crust formation at the product surface [36]. As a consequence, concentration differences (or different local supersaturation values) prevail for different heights inside the film. The deviation between the average water content and the local water content at the surface increases with faster drying. The different nucleation behavior in terms of the onset and rate depending on the humidity of the drying air can yet be explained by these different vertical concentration differences prevailing in the film at different stages of drying, as illustrated schematically in Figure 8.

If the water content of the film has not fallen below a critical water content (X_crit_) over the films’ entire height, crystallization does not occur, even though saturation has already been exceeded (X_sat_). This is shown in Figure 8 for films dried at different air humidities at an average water content, ${\bar{X}}_{1}$.

An early onset of nucleation under anhydrous drying air conditions (blue lines; 0% RH) exhibiting fast drying kinetics is caused by an earlier attainment of a nucleation-favoring water content, X_crit_ (see Figure 8 at an average water content, ${\bar{X}}_{2}$)—respectively, the boundary of the metastable zone—in the uppermost slab of the film. At the same time, the average water content ${\bar{X}}_{2}$ of the film due to the less concentrated, deeper layers is still comparatively high at this point of drying and lies inside the metastable zone. With slower drying due to higher air humidity (orange line; 15% RH), critical water contents in the film surface, which enable nucleation, are not reached at the same average water content (${\bar{X}}_{2}$), as more diffusive concentration equalization is possible within the thin film. Crystallization starts delayed at an average water content below ${\bar{X}}_{2}$. At high humidities (green line, 30% RH), the thin film exceeds the saturation water content (X_sat_), but the critical water content is never reached in the upper layers as the drying equilibrium (X_eq,30%_) is achieved above X_crit_ with an amorphous film as a result. These assumptions are valid for our experiments as temperature in the film is comparable throughout all experiments of different drying air humidity: crystallization commences in the second drying phase and drying rate manipulation is achieved solely by a higher air humidity.

At the same time, this approach explains the experimental observation of the considerably higher nucleation rates directly after the onset as well as the (sharp) increase in the rate (see Figure 6 and Figure 7) when drying with moderate air humidity. This is pointed out in the comparison of moderately moist air (orange line) with dry air (blue line) at the same mean water content of ${\bar{X}}_{3}$ in Figure 8: while the water content is falling below X_crit_ (or, respectively, the metastable zone is surpassed), values favorable for nucleation are reached simultaneously in a comparatively higher percentage of the film thickness (h − h_1_), whereas with fast and harsh drying (by anhydrous drying air), ideal supersaturation values for nucleation are only present in a thinner slab of the film (h − h_2_). This leads to a lower nucleation-prone volume share and thus lower nucleation rates for the harshly dried film. In the same way, this theory explains the steeper drop in the nucleation rate with moderately humid air (15% RH) at the end of drying and low mean water contents due to a simultaneous reaching of the viscosity critical for nucleation, involving, respectively, less difference between the film surface and deeper layers.

### 3.5. Crystallization under Decreasing In Situ Humidity

The previously discussed experimental results about the nucleation rates (see Figure 6 and Figure 7) suggest that the nucleation rates are also water content-dependent in the highly concentrated, amorphous state, similar to those in slightly supersaturated solutions [26]. Ergo, as drying progresses and crust formation (respectively, the formation of a higher film-internal concentration difference between the surface and deeper layers) occurs, different nucleation rates are simultaneously present in the thin film. Hence, the observed nucleation rate cannot be used to infer the true rate at the measured, average film concentration; rather, this is the result of the different nucleation rates prevailing in the film at this drying state.

**Figure 9:**
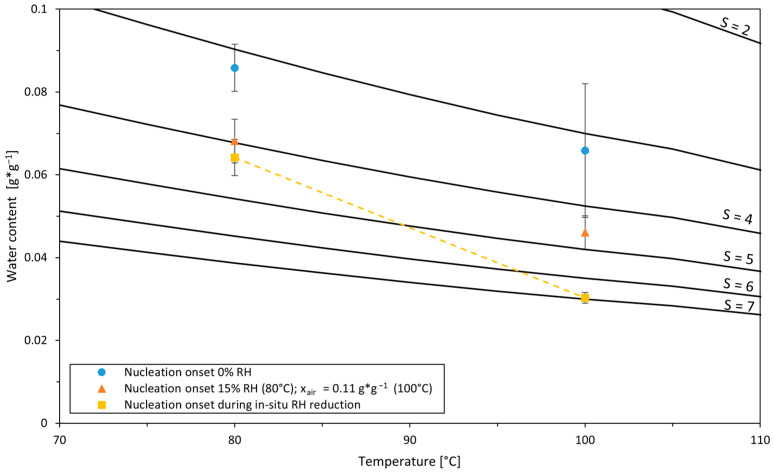
Nucleation onset for different drying air temperatures and relative humidities (0 and 15% RH for 80 °C; humidity ratio of 0 and 0.11 g*g^−1^ for 100 °C) as well as for experiments, where drying air humidity was incrementally decreased in situ. Error bars denote mean absolute errors. The dashed line illustrates the shift of the metastable zone and serves to guide the eye.

In order to substantiate the assumption that the films’ internal, vertical concentration profile plays a decisive role in crystallization behavior (nucleation onset and rate), further experiments were carried out for 80 °C, whereby the temperature, i.e., the saturation concentration in the second drying stage, was kept constant and the humidity of the drying air was slowly reduced in situ. It was assumed that the vertical concentration profile in the film is minimized, leading to lower deviations of the water content between the surface and deeper film layers near the experimentally measured $\bar{X}$. Also, such experiments were carried out for 100 °C as well as experiments at 100 °C and constant humidity. Figure 9 shows the water content at the nucleation onset of the experiments with constant and reduced air humidity in relation to the corresponding supersaturation levels.

At the point of onset, for the drying experiments with constantly anhydrous air, the thin film dried at 80 °C exhibits a very similar supersaturation of S = 3.16 compared to that dried at 100 °C with S = 3.19. However, this does not apply to the experiments with slightly humid air (15% RH for 80 °C and x_air_ = 0.11 g*g^−1^ for 100 °C, respectively). Here, the average supersaturation of the film at 100 °C at the onset of crystallization with S = 4.56 is remarkably higher than the supersaturation at 80 °C with S = 3.98. The films dried with a stepwise in situ reduction in drying air humidity at 80 °C were allowed to dry to a supersaturation of S = 4.23 ± 0.28 (corresponding water content X = 0.064 ± 0.004 g*g^−1^) before nucleation occurred. For films dried in increments at 100 °C, this was even possible up to a supersaturation of S = 6.95 ± 0.29 (X = 0.030 ± 0.001 g*g^−1^). Thus, for both temperatures, a considerably higher concentration was achieved until the nucleation onset by stepwise drying than for drying at constant humidity, although the effect was much more pronounced at 100 °C.

With steps of constant air humidity and the resulting slow overall drying kinetics in the range relevant for crystallization, it was assumed that sufficient diffusive equalization to an approximately homogeneous, internal film concentration was possible. Furthermore, any low nucleation rate could be detected by leaving sufficient time after reaching each equilibrium water content. This ensures that relevant rates, which could play a role in the process, are recognized. The retarded nucleation onset in incrementally dried films underpins the relevance of the concentration distribution within the film in general. Furthermore, at the temperatures investigated, it was thus possible to define the probable range of the previously mentioned critical water contents for crystallization (X_crit_) or, respectively, the border of the metastable zone, illustrated in Figure 9 by the dashed, yellow line. It is interesting to note that the metastable zone is significantly wider at 100 °C than at 80 °C. The result is also in agreement with the generally lower nucleation rates at 100 °C (see Figure 7) compared with the rates at 80 °C for the experiments at constant drying air humidity: the relative distance between the water content range in which nucleation occurred at constant relative humidity and the boundary of the metastable zone was greater for the experiments at higher temperature. It is therefore reasonable to assume that the nucleation rate, as soon as the films’ surface exceeds the metastable zone, will react less sensitively during ongoing drying if the metastable zone is wider in relation to the water content.

With precise knowledge about the width of the metastable zone and the growth and nucleation behavior over the whole drying progress, it is theoretically possible to tailor the product properties more specifically regarding the physical state by drying parameter manipulation: slow drying to the boundary of the metastable zone can suppress nucleation (which is a basis for crystallization) and maximize the concentration in the amorphous state. In the case of sucrose or sucrose-rich products, for example, the glass transition temperature T_g_ of the concentrated aqueous system at maximum concentration before the nucleation onset is around 38 °C for drying at 100 °C and around 16 °C for drying at 80 °C (derived by the Gordon–Taylor equation, k = 4.7; T_g,Sucrose_ = 62 °C and T_g,Water_ = −135 °C from [37]). Consequently, a product that is stable under ambient conditions could be produced. On the other hand, with fast, high-temperature drying, a high degree of crystallization could be achieved in the product despite low nucleation rates and a broad metastable zone, for example, by enabling early and high crystal growth rates combined with seeding (resp. heterogeneous nucleation) or similar. The same could be achieved by suitable temperature control in two steps using an initially low drying temperature, whereby crystallization is induced early by the existence of a narrow metastable zone in this temperature range. Subsequent rapid growth combined with shortened drying time could then be achieved at high temperatures.

## 4. Conclusions

The results of the investigation clarify in detail the progress of the formation of sucrose crystallinity depending on the process conditions of drying temperature and humidity. The experiments show that combined thin-film drying and polarized light imaging is well suited for experimentally observing the nucleation onset, nucleation rates, and crystal growth rates separately from each other under defined drying conditions and during the whole drying process. For the first time, crystallization behavior was investigated in this differentiated way and under precisely defined drying conditions with regard to temperature and humidity.

Higher drying temperatures increased crystal growth; however, temperatures above 100 °C caused the growth rate to stagnate due to the high viscosity of the system.

The interplay between drying air temperature and humidity governs the nucleation behavior, primarily by controlling the film’s internal, vertical concentration profile. Low humidity creates a steep concentration gradient between the surface and deeper layers of the film, promoting earlier nucleation in the higher concentrated surface. In contrast, higher humidity levels (15% RH) allow for more uniform moisture distribution, delaying nucleation but enabling a considerably higher (approximately doubled) nucleation rate since the concentration range favorable for nucleation is reached at the same time in a broad, higher film fraction. Excessively high humidity, such as 60% RH, completely inhibited nucleation as the supersaturation at moisture equilibrium is too weak to achieve relevant nucleation in the time scale of thin-film drying. The results narrow down the existence of relevant stages for inline crystallization. The range is bounded by the nucleation onset on the one hand and inhibition of growth and nucleation on the other, which can be influenced by the drying kinetics. The present work provides an advanced experimental insight into the crystallization behavior and the causes of crystallinity of final products by differentiating between the nucleation onset, nucleation rate, and crystal growth as a function of drying progress.

In summary, a deep mechanistic understanding of the formation of the physical state during convective drying is gained and the findings offer insights into manipulating the drying parameters to achieve desired product crystallinity.

## Data Availability

The data that support the findings of this study are available within the article and its Appendix A. Further data are available from the corresponding author upon reasonable request.

## Appendix A

**Table A1:** Drying conditions used during the experiments. Relative humidity values and air humidity ratios x_air_ are derived by the Antoine Equation [38]. As the relative humidity scale becomes misleading at temperatures at and above 100 °C and atmospheric pressure, the air humidity ratio xair, defined as the vapor mass per unit mass of dry air, is used for the experiments at 100 °C.

Air Temperature T [°C]	Relative Humidity RH [%]	Humidity Ratio x_air_ [g*g^−1^]	Air Velocity v_air_ [m*s^−1^]
80	0	0	0.3
80	15	0.047	0.3
80	30	0.101	0.3
80	60	0.242	0.3
100	--	0	0.3
100	--	0.111	0.3
100	--	0.270	0.3
100	--	0.945	0.3
